# Karyological study of *Amphisbaena ridleyi* (Squamata, Amphisbaenidae), an endemic species of the Archipelago of Fernando de Noronha, Pernambuco, Brazil

**DOI:** 10.1590/S1415-47572010005000009

**Published:** 2010-03-01

**Authors:** Marcia Maria Laguna, Renata Cecília Amaro, Tamí Mott, Yatiyo Yonenaga-Yassuda, Miguel Trefaut Rodrigues

**Affiliations:** 1 Departamento de Genética e Biologia Evolutiva, Instituto de Biociências, Universidade de São Paulo, São Paulo, SPBrazil; 2 Departamento de Zoologia, Instituto de Biociências, Universidade de São Paulo, São Paulo, SPBrazil; 3 Instituto de Biociências, Programa de Pós Graduação em Ecologia e Conservação da Biodiversidade, Universidade Federal do Mato Grosso, Cuiabá, MTBrazil

**Keywords:** *Amphisbaena ridleyi*, karyotype, Fernando de Noronha, Ag-NOR, FISH with telomeric probes

## Abstract

The karyotype of *Amphisbaena ridleyi*, an endemic species of the archipelago of Fernando de Noronha, in State of Pernambuco, Brazil, is described after conventional staining, Ag-NOR impregnation and fluorescence *in situ* hybridization (FISH) with a telomeric probe. The diploid number is 46, with nine pairs of macrochromosomes (three metacentrics, four subtelocentrics and two acrocentrics) and 14 pairs of microchromosomes. The Ag-NOR is located in the telomeric region of the long arm of metacentric chromosome 2 and FISH revealed signals only in the telomeric region of all chromosomes. Further cytogenetic data on other amphisbaenians as well as a robust phylogenetic hypothesis of this clade is needed in order to understand the evolutionary changes on amphisbaenian karyotypes.

Amphisbaenians, or worm lizards, are a monophyletic group of squamates mostly distributed nowadays in Africa and South America (Gans, 1990, 2005; Kearney, 2003; Kearney and Stuart, 2004; Macey *et al.*, 2004). Due to their fossorial lifestyle and the consequent challenge for collecting them, the group is probably the least-studied group of squamates and many aspects of its biology remain enigmatic.

Although a phylogenetic hypothesis based on morphological and molecular characters for the group was only recently proposed (Kearney, 2003; Kearney and Stuart, 2004; Macey *et al.*, 2004; Vidal *et al.*, 2008), karyological studies on amphisbaenians date back from the 1960's. The karyotypes of 35 out of the 190 recognized amphisbaenian species have been described, mostly including only data on diploid number and chromosomal morphology (Table 1). Amphisbaenian karyotypes present variable diploid number and morphology with distinctive macro and microchromosomes. Diploid numbers range from 2n = 26 in *Amphisbaena dubia* and *Anops kingi* to 2n = 50 in *Amphisbaena leberi* and *A. innocens* (Huang and Gans, 1971; Beçak *et al.*, 1971a, 1972; Cole and Gans, 1987). This variability is in strong contrast with the conserved karyotype composed by 36 chromosomes (12M + 24m) found in many groups of lizards and considered as the primitive karyotype within Squamata (Olmo, 1986). Except for the study of Hernando (2005) describing the localization of nucleolar organizer regions (NORs) in four South American species, all chromosomal studies in amphisbaenians only presented conventional staining data.

Herein we describe the chromosome constitution of *Amphisbaena ridleyi,* a species endemic to the oceanic archipelago of Fernando de Noronha, Pernambuco, Brazil. Although this species resembles some African members in some external attributes, molecular data indicate that it is closely related to the South American genus *Amphisbaena* (Gans, 1963; T. Mott, unpublished data). Karyotypic data presented here support the idea that amphisbaenian karyotypes are highly variable and might assemble phylogenetically informative characters. This information allied to phylogenetic hypotheses of amphisbaenian relationships would help to understand the chromosome evolution in this interesting group of fossorial squamates.

Three individuals of *Amphisbaena ridleyi* were collected by two of us (TM, MTR; IBAMA permit number 02010.000240/2007-03), one male (MZUSP 98333) and one female (MZUSP 98335) from the Ilha Rata (3°48'47.6” S, 32°23'21.5” W) and one female (MZUSP 98338) from the Ilha Fernando de Noronha (3°51'21.2” S, 32°26'31.5” W), both in the archipelago of Fernando de Noronha, Pernambuco, Brazil. The animals were brought alive to the Laboratório de Citogenética de Vertebrados, Departamento de Genética e Biologia Evolutiva, Instituto de Biociências, Universidade de São Paulo, Brazil and after chromosomal preparations were made, the specimens were deposited in the herpetological collection of Museu de Zoologia, Universidade de São Paulo.

The animals were injected with colchicine, according to routine techniques (Kasahara *et al.*, 1987), and chromosomal spreads were obtained from the liver. The diploid number and the localization of Ag-NORs were established after conventional staining and silver staining impregnation (Howell and Black, 1980), respectively. Fluorescence *in situ* hybridization (FISH) was performed using the Telomere PNA FISH Kit/Cy3 (DAKO, code No. K 5326), according to manufacturer's instructions. FISH signals were visualized using a Zeiss Axiophot microscope equipped with a FITC filter using the softwares Ikaros & Isis v. 5.0 (Zeiss).

*Amphisbaena ridleyi* from the Ilhas Rata and Fernando de Noronha had similar karyotype numbers composed by 46 chromosomes, with 9 pairs of macrochromosomes and 14 pairs of microchromosomes (2n = 46, 18M+28m) (Figure 1). The macrochromosomes are three metacentric pairs (1, 2 and 4), two acrocentric pairs (8 and 9) and four subtelocentric pairs (3, 5, 6 and 7), although in some metaphases the short arms of some of these chromosomes was extremely reduced. There was not enough resolution to morphologically identify the 14 pairs of microchromosomes. No secondary constrictions or heteromorphic sex chromosomes were observed.

The karyotype described for *A. ridleyi* (2n = 46, 18M + 28m) is unique among its congeners (Huang *et al.*, 1967; Huang and Gans, 1971; Beçak *et al.*, 1972, 1973a; Cole and Gans, 1987; Hernando, 2005). Furthermore, the comparison of the karyotype of *A. ridleyi* with those of other amphisbaenian genera with the same diploid number, such as *Bipes canaliculatus, Bipes tridactylus* (22M + 24m)*, Mesobaena huebneri* (24M + 22m) and *Rhineura floridana* (26M +20m), revealed that the number of macro and microchromosomes and the number of biarmed chromosomes were very distinct among different genera, (Table 1) (Matthey, 1933; Huang *et al.*, 1967; Macgregor and Klosterman, 1979; Cole and Gans, 1987).

Despite the fact that only 20% of amphisbaenian species have had their karyotypes studied, a great variability of diploid numbers has been observed. There are 77 described species of *Amphisbaena* (Gans, 2005; Mott *et al.* 2008, 2009) from which 18 had their karyotypes described, including *A. ridleyi* from the present study. This is the genus that exhibits the higher variability in chromosome number and morphology, including all the range of variation found in amphisbaenians, such as 2n = 26 (14M + 12m) in males and 2n = 25, 26, 27 and 28 in females of *A. dubia*; 2n = 30 (12M + 18m) in *A. angustifrons*, *A. darwini*, *A. heterozonata*, *A. hiata*, *A. trachura*; 2n = 36 (12M + 24m) in *A. caeca*, *A. fenestrata*, *A. manni*, *A. xera*; 2n = 38 (22M + 16m) in *A. alba*; 2n = 40 (18M + 22m) in *A. mertensi*; 2n = 44 in *A. camura* (24M + 20m) and *A. vermicularis* (22M + 22m); 2n = 48 (22M + 26m) in *A. fuliginosa* and 2n = 50 (22M + 28m) in *A. leberi* and *A. innocens* (Table 1). Probably fusion/fission rearrangements occurred in the karyotypic diversification of amphisbaenians (Cole and Gans, 1987; Hernando, 2005), but the number of taxa studied and the absence of differential staining do not allow more detailed hypotheses on the karyotypic evolution of this group.

Some karyotypes reported in the literature do not allow to determine the fundamental number due to the difficulty of identifying the morphology of the microchromosomes. The species *A. dubia* showed an intraindividual variation of the diploid number, involving macro-and microchromosomes, and the authors suggested that this would be due to fusions/fissions of microchromosomes (Beçak *et al.* 1971a, 1972). However, the polymorphism detected in *A. dubia* should be viewed with reservations due to the low quality of the chromosome preparations.

The Ag-NORs of all specimens of *A. ridleyi* were located in the telomeric region of the long arm of the metacentric pair 2 (Figure 2) in 17 metaphases on specimens from Ilha Rata and in 13 metaphases on the specimen from Fernando de Noronha, differing from all four South American amphisbaenian species previously studied (Hernando, 2005). In *Leposternum microcephalum* (2n = 34, 12M + 22m), Ag-NORs were detected in the telomeric region of the long arm of pair 3; in *A. hiata* (2n = 30, 12M +18m) it was located in the subterminal portion of the short arm of pair 4; in *A. mertensi* (2n = 40, 18M + 22m) a medium acrocentric macrochromosome was the Ag-NOR-bearing pair, and in *A. heterozonata* (2n = 30, 12M + 18m), Ag-NORs were found either in pair 2 or in pairs 1, 3 and 4 (Hernando, 2005).

Fluorescence *in situ* hybridization using the (TTAGGG)_n_ sequence detected signals on the telomeric regions of all chromosomes of *A. ridley* (Figure 3). Some of the signals were tiny and sometimes it was difficult to visualize them in the photographs. Despite the small number of studies using fluorescence *in situ* hybridization in Squamata, different patterns of distribution of telomeric sequences were observed. In *Leposoma scincoides* (Gymnophthalmidae), *Polychrus marmoratus* (Polychrotidade) and *Phrynosoma cornutum* (Phrynosomatidae) only telomeric signals were detected, while the chromosomes of *Cnemidophorus sexlineatus*, *C. guturalis* (Teiidae), *Scelophorus olivaceus*, *Cophosaurus texanus* (Phrynosomatidae), *Gonatodes taniae* (Gekkonidae), *Leposoma guianense*, *Leposoma osvaldoi* (Gymnophthalmidae) and *Polychrus acutirostris* (Polychrotidade) presented additional interstitial telomeric sites (Meyne *et al.*, 1989, 1990; Schmid *et al.*, 1994; Pellegrino *et al.*, 1999; Bertolotto *et al.*, 2001). The exclusive telomeric pattern observed in *A. ridleyi* is the first report of FISH for amphisbaenids.

The New World amphisbaenids form a monophyletic group within the paraphyletic radiation of African amphisbaenids (Kearney and Stuart, 2004; Vidal *et al.*, 2008). African members of Amphisbaenidae show a lower range of variation in diploid number, like *Cynisca leucura* (2n = 30) and *Geocalamus acutus* (2n = 38) (Huang and Gans, 1971), when compared to South American congeners. Nevertheless, a more complete taxonomic sampling, including cytogenetic data with differential staining analyses, is needed in order to obtain a better picture of karyotype evolution in amphisbaenids. Despite the scarce information about Ag-NORs location on amphisbaenian karyotypes, the preliminary data available suggest that this marker is phylogenetically informative. We strongly recommend that further studies on amphisbaenian karyotypes include this information.

**Figure 1:**
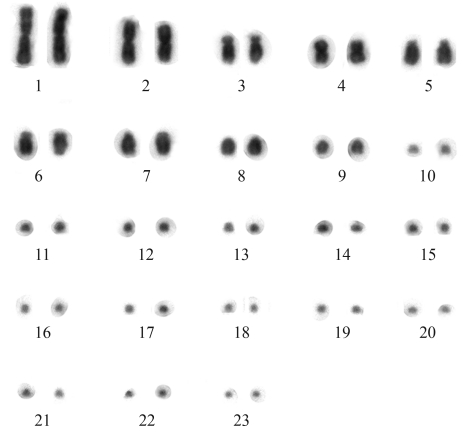
Conventionally stained karyotype of *Amphisbaena ridleyi*, female, 2n = 46 (18M + 28m), from Fernando de Noronha, Pernambuco, Brazil.

**Figure 2:**
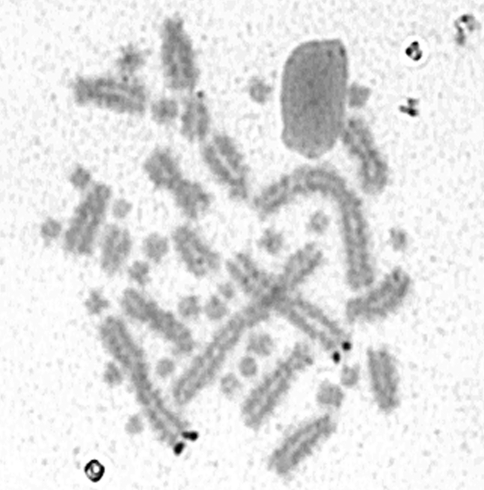
Incomplete metaphase after silver staining showing the Ag-NORs on the telomeric region of chromosome 2 of *Amphisbaena ridleyi* from Fernando de Noronha (Pernambuco, Brazil).

**Figure 3:**
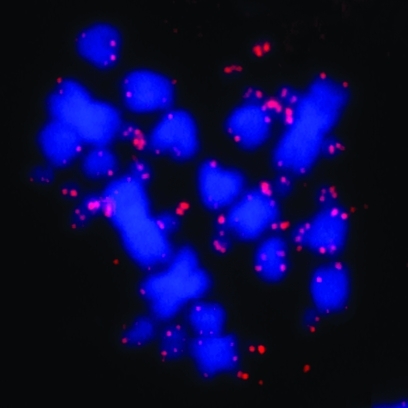
Distribution of the (TTAGGG)n sequence in chromosomes of*Amphisbaena ridleyi*, from Fernando de Noronha (Pernambuco, Brazil).

## Figures and Tables

**Table 1:** Chromosomal revision of amphisbaenians, with descriptions of diploid number (2n), fundamental number (FN), number and morphology of macrochromosomes, number of microchromosomes, references and occurrence of species.

Species	2n	Macro (n. biarmed, n. uniarmed)	micro	FN	Reference^1^	Occurrence
Amphisbaenidae						
*Amphisbaena alba*	38	22 (14, 8)	16	64	4, 5, 6, 7	South America
*Amphisbaena angustifrons*	30	12 (12, 0)	18	42	2	South America
*Amphisbaena caeca*	36	12 (12, 0)	24	48	2	Central America
*Amphisbaena camura*	44	24 (4, 20)	20	48-50^2^	2	South America
*Amphisbaena darwini*	30	12 (12, 0)	18	46	2	South America
*Amphisbaena dubia*	25, 26, 27, 28	15 (12, 3), 14 (12, 2), 13 (12, 1), 12 (12, 0)	10, 12, 14, 16	-	3, 6	South America
*Amphisbaena fenestrata*	36	12 (12, 0)	24	52-56^2^	5	Central America
*Amphisbaena fuliginosa*	48	22 (6, 16)	26	60	5	South America
*Amphisbaena heterozonota*	30 30	12 (12, 0) 12 (12, 0)	18 18	46 60	2 12, 13	South America
*Amphisbaena hiata*	30	12 (12, 0)	18	60	12, 13	South America
*Amphisbaena innocens*	50	22 (8, 14)	28	-	5	Central America
*Amphisbaena leberi*	50	22 (8, 14)	28	-	11	Central America
*Amphisbaena manni*	36	12 (12, 0)	24	-	5	Central America
*Amphisbaena mertensi*	40	18 (6, 12)	22	-	12	South America
*Amphisbaenaridleyi*	48	18 (14, 4)	28	-	Present work	South America
*Amphisbaena trachura*	30	12 (12, 0)	18	46	2	South America
*Amphisbaena vermicularis*	44	22 (2, 20)	22	46	7, 8	South America
*Amphisbaena xera*	36	12 (12, 0)	24	48	2	Central America
*Anops kingi*	26	12 (12, 0)	14	-	5	South America
*Chirindia langi*	30 34	12 (12, 0) 12 (12, 0)	18 22	- -	5	Africa
*Chirindia* sp	30 32	12 (12, 0) 12 (12, 0)	18 20	46-50^2 ^-	5	Africa
*Cynisca leucura*	30 32	12 (12, 0) 12 (12, 0)	18 20		5	Africa
*Geocalamus acutus*	38	14 (10, 4)	24	-	5	Africa
*Leptosternon microcephalum*	34 32 34	12 (12, 0) 12 (12, 0) 12 (2,22)	22 20 22	48 44 46	4, 6 2 2, 12, 13	South America
*Mesobaena huebneri*	46	24 (2, 22)	22	-	10	South America
*Monopeltis capensis*	34	12 (12, 0)	22	62	5	Africa
*Zygaspis quadrifrons*	36 36	12 (12, 0) 12 (12,0)	24 24	50 72	2 5	Africa
*Zygaspis violacea*	36	12 (12, 0)	24	-	5	Africa

Bipedidae						
*Bipes biporus*	40 42	20 (20, 0) 20 (20, 0)	20 22	60 66	2 5, 9, 11	North America
*Bipes canaliculatus*	46 46	22 (16, 6) 22 (20, 2)	24 24	- -	9 11	North America
*Bipes tridactylus*	46	22 (18, 4?)	24	-	11	North America

Blanidae						
*Blanus cinereus*	32	12 (12, 0)	20	44	2	Europe
*Blanus strauchi*	32	12 (12, 0)	20	44	2	Europe

Rhineuridae						
*Rhineura floridana*	46 44	20 (2, 18) 24 (16, 8)	26 20	- 54-56^2^	1 2	North America

Trogonophiidae						
*Diplometopon zarudnyi*	36	12 (12, 0)	24	52	2, 10	Africa
*Trogonophis elegans*	36	12 (12, 0)	24	48	2	Africa

^1^: 1. Matthey (1933); 2. Huang *et al.* (1967); 3. Beçak *et al.* (1971a); 4. Beçak *et al.* (1971b); 5. Huang and Gans (1971); 6. Beçak *et al.* (1972); 7. Beçak *et al.* (1973a); 8. Beçak *et al.* (1973b); 9. Macgregor and Klosterman (1979); 10. Branch (1980); 11. Cole and Gans (1987); 12. Hernando (2005); 13. Hernando and Alvarez (2005). ^2^: The variation of FN, according to the authors, is due to the difficulty in determining microchromosome morphology.
